# Behavioral self-regulation for weight loss in young adults: a randomized controlled trial

**DOI:** 10.1186/1479-5868-6-10

**Published:** 2009-02-16

**Authors:** Jessica Gokee-LaRose, Amy A Gorin, Rena R Wing

**Affiliations:** 1 Brown Medical School, The Miriam Hospital, Weight Control and Diabetes Research Center, Providence, Rhode Island, USA; 2 University of Connecticut, Department of Psychology, Center for Health, Intervention, and Prevention, Storrs, Connecticut, USA

## Abstract

**Objective:**

To determine the feasibility of recruiting and retaining young adults in a brief behavioral weight loss intervention tailored for this age group, and to assess the preliminary efficacy of an intervention that emphasizes daily self-weighing within the context of a self-regulation model.

**Methods:**

Forty young adults (29.1 ± 3.9 years, range 21–35, average BMI of 33.36 ± 3.4) were randomized to one of two brief behavioral weight loss interventions: behavioral self-regulation (BSR) or adapted standard behavioral treatment (SBT). Assessments were conducted at baseline, post-treatment (10 weeks), and follow-up (20 weeks). Intent to treat analyses were conducted using general linear modeling in SPSS version 14.0.

**Results:**

Participants in both groups attended an average of 8.7 out of 10 group meetings, and retention rates were 93% and 88% for post-treatment and follow-up assessments, respectively. Both groups achieved significant weight losses at post-treatment (BSR = -6.4 kg (4.0); SBT = -6.2 kg (4.5) and follow-up (BSR = -6.6 kg (5.5); SBT = -5.8 kg (5.2), *p *< .001; but the interaction of group × time was not statistically significant, *p *= .84. Across groups, there was a positive association between frequency of weighing at follow-up and overall weight change at follow-up (*p *= .01). Daily weighing was not associated with any adverse changes in psychological symptoms.

**Conclusion:**

Young adults can be recruited and retained in a behavioral weight loss program tailored to their needs, and significant weight losses can be achieved and maintained through this brief intervention. Future research on the longer-term efficacy of a self-regulation approach using daily self-weighing for weight loss in this age group is warranted.

**Clinical Trials Registration:**

# NCT00488228

## Background

Young adults are increasingly at risk for obesity. Data indicate that more than half of women and men between the ages of 20 and 39 are overweight or obese [1]. Young adults also experience the greatest rate of weight gain, averaging 1 to 2 pounds per year, with significant increases during their early to mid-twenties [2,3]. Further, data indicate that the largest weight gains occur among those young adults who are already overweight [3], and young adults who are overweight initially also are at greatest cardiovascular risk due to weight gained during these years [2-4]. Despite the problem of overweight and obesity in young adults, such individuals are underrepresented in standard behavioral weight loss programs, and on average, those who do enroll achieve less than half the weight loss achieved by older adult participants [5]. In fact, recent reanalysis of data from two ongoing adult behavioral weight loss trials demonstrated that only 7% of enrolled participants were between 18 and 35; and at 6 months, young adults in these trials has lost an average of only 3.7 kg, compared to an average of 8.0 kg achieved by older adults in these same studies [5]. To date, no treatment programs have specifically targeted young adults for weight loss, and little is known about the type of approach best suited for this critical period in the developmental lifespan.

One of the simplest strategies that holds potential to reverse the weight gain commonly associated with this period is teaching young adults to weigh themselves frequently and use this information to make adjustments in their behavior as needed. Frequent self-weighing has been associated with positive outcomes in weight gain prevention, weight loss, and weight maintenance studies [6-9]. To date, however, frequency of weighing has not been directly manipulated within a randomized clinical trial for weight loss, and doing so within a self-regulation framework may improve current treatment efforts. Specifically, self-regulation in this context includes *self-observation *(i.e., regular weighing), *self-evaluation *(i.e., comparing one's weight to desired weight range), and *self-reinforcement *(i.e., reinforcing efforts to lose weight or maintain weight) or *adjustments in behavior *(i.e., making changes to calorie or exercise prescription).

Self-regulation programs of a similar nature have been effective in the management of diabetes [10], as well as weight loss maintenance [9]. A self-regulation program using daily weighing may be particularly beneficial in improving weight loss efforts with young adults, who, as a group struggle with self-regulation across a variety of health-related behaviors. For example, extant research indicates that cigarette smoking, binge drinking, and heavy alcohol use all peak in young adulthood [11-13]. As in weight control, self-monitoring of these behaviors is related to decreased use [14,15], and researchers have used self-regulation theory as a framework for these findings [15]. Directly relevant to the self-regulation of weight, as adolescents become young adults, they are more likely to eat fast food and decrease their exercise [16]. Daily self-weighing provides a mechanism to allow young adults to see how their behaviors (e.g., alcohol or fast food intake) impact their weight and would enable them to react with appropriate behavior changes before weight gain accumulates over time. It is well established that self-monitoring is predictive of weight loss [17], but it is also well known that self-monitoring declines over time [18]. Daily weighing may be more easily maintained than traditional forms of self-monitoring (i.e., calorie and fat intake), and therefore may be appealing to young adults in the midst of significant life transitions and with considerable time demands.

Despite the potential benefits associated with daily self-weighing, some researchers have argued that frequent weighing may result in an increased risk of developing eating disorders or in negative effects on mood [19,20]. However, there are few data to support these concerns [21], and the studies that have raised these concerns have been conducted with adolescent samples, not adults [20,22,23]. Rather, available data provide no evidence of adverse effects of daily weighing within an adult population [24]. However, this has not been evaluated specifically in a young adult sample, in which the development and/or onset of disordered eating behaviors may be more likely [25].

General goals of the current pilot study were to assess the feasibility of recruiting and retaining overweight and obese young adults in a behavioral weight loss program, and to evaluate the potential to achieve significant weight losses through a brief intervention. More specifically, we sought to determine whether young adults would adhere to different self-weighing prescriptions, whether daily weighing and self-regulation would improve weight loss outcomes, and whether untoward effects of daily weighing would be observed. We randomized participants to one of two groups: a behavioral self-regulation (BSR) program that used daily self-weighing, or a tailored version of standard behavioral treatment (SBT) that promoted weekly weigh-ins at group only. Both groups were active treatments that taught the same core behavioral strategies, provided identical calorie and exercise prescriptions, and limited enrollment to young adults between 21 and 35. Further, lessons in both groups were tailored to specific problem areas facing this age group (e.g., fast food, alcohol, time management). We hypothesized that participants in both treatment arms would adhere to their self-weighing prescriptions, and that both arms would produce significant weight losses at post-treatment. We predicted that the self-regulation condition that emphasized daily weighing would lead to better longer-term weight losses once treatment was discontinued.

## Methods

### Participants

To participate, individuals had to be between 21 and 35 years old with a BMI between 27–40 kg/m^2^, and could not have a history of an eating disorder or substance abuse, be participating in another weight loss program, have lost ≥ 5% of their body weight within 6 months, or be unable to participate in unsupervised exercise. For the current study we chose to exclude individuals between 18 and 20 years of age given the high proportion of college students in this age range and 1) the needs of college students may be distinct from other young adults and this group is often studied separately; 2) there are potential concerns about daily weighing and eating disorders in this group; and 3) due to ethical considerations related to the discussion of alcohol in this program with individuals who are under the legal drinking age. We selected 35 years of age as our upper limit for enrollment because data indicate that in the U.S. among adults between 25 and 74, weight gain over 10 years was highest at ages 25–35.

**Figure 1:**
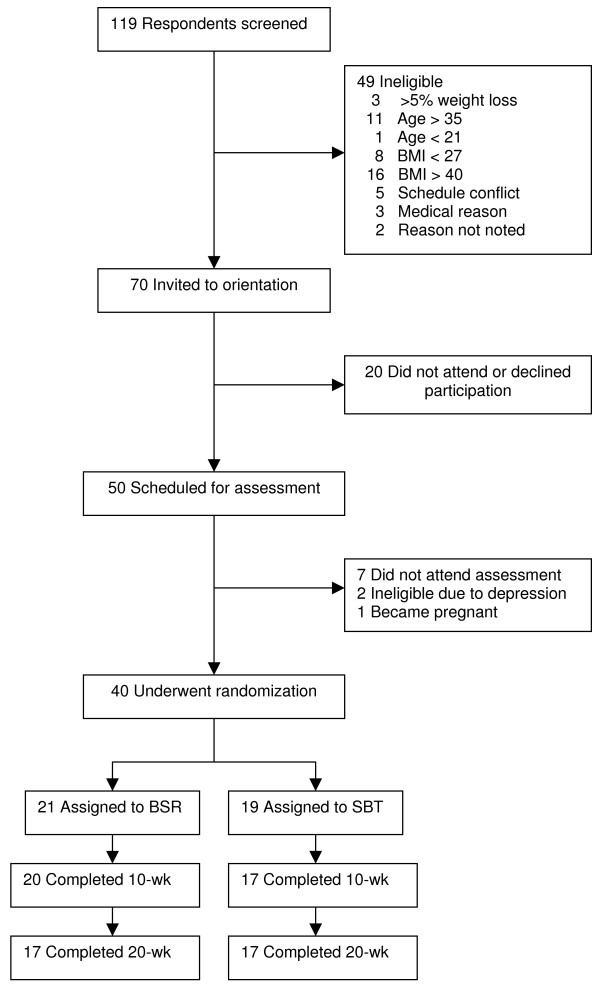
**Study Enrollment and Retention**.

A total of 119 participants were screened by phone to determine preliminary eligibility (see Figure 1 for details). A total of 40 young adults were randomized to treatment (35 female and 5 male); on average, they were 29.1 ± 3.9 years old with a BMI of 33.36 ± 3.4 kg/m^2 ^and a mean baseline weight of 82.3 ± 12.6 kg. Participants were 19.5% Hispanic, 75% Caucasian, 7.5% Black/African-American, and 17.5% Biracial or other. Baseline characteristics did not differ between the treatment conditions.

### Procedure

Participants were recruited through advertisements in the local media, email blasts, and flyers posted in the Providence area. Interested participants contacted us and completed a brief phone screen to determine preliminary eligibility. Those who were eligible and remained interested were invited to attend a small group orientation visit where the details of the study were described and informed consent was obtained. Participants who consented completed baseline assessments visits at which measures of height and weight were taken, and study questionnaires were completed. Those participants who remained eligible were then randomly assigned to treatment groups.

### Design

Participants were randomly assigned to one of two groups: a behavioral self-regulation condition (BSR) or a tailored version of standard behavioral treatment (SBT). Both groups met weekly for 10 weeks, followed by a booster session at week 14. Assessments took place at baseline, post-treatment (i.e., 10 weeks) and at a follow-up visit at 20 weeks. Additionally, at the follow-up assessment visit participants were asked to complete anonymous feedback forms about their experience in the Live Well program, as well as their preferences for format and duration of a weight loss program. Participants were paid $20 for completing the post-treatment assessment visit, and an additional $20 for the follow-up visit. We certify that all applicable institutional and governmental regulations concerning the ethical use of human volunteers were followed during this research. This study was approved by the Institutional Review Board at the Miriam Hospital in Providence, Rhode Island.

### Common Treatment Components across Both Groups

#### Contact

Treatment consisted of 10 weekly group meetings, each 60 minutes in length, with an optional booster session at week 14.

#### Dietary goals

Daily calorie goals were based upon weight at study entry; individuals with an entry weight ≤ 91 kg were prescribed 1200 kcals/day and those with an entry weight of > 91 kg were prescribed 1500 kcals/day. Participants also were given a target of no more than 20–30% calories from fat.

#### Exercise goals

Research has demonstrated that young adults, in particular, are at risk for decreasing their physical activity. In fact, rates of physical activity appear to decline significantly during the transition from adolescence to adulthood [26]. Therefore, the importance of physical activity was stressed throughout the current program. Participants were instructed to gradually increase their physical activity until they were active at least 40 minutes a day 5 times per week. To minimize barriers for physical activity, participants were encouraged to do brisk walking and were allowed to accumulate time spent being physically active by engaging in multiple short bouts (e.g., at least 10 minutes in length).

#### Behavior modification skills

Participants were instructed in core behavioral and cognitive skills, which form the basis of behavioral obesity treatments and are used to help participants implement changes in eating and activity behaviors (e.g., self-monitoring, stimulus control, goal setting, relapse prevention).

#### Tailoring of session content

Lessons were adapted to place greater emphasis on problematic behaviors of particular relevance to this age group. For example, fast food consumption increases as adolescents become young adults [27], and fast food consumption and change in fast food consumption is related to the magnitude of weight gain and obesity in this age group [28,29]. Also, data indicate that high percentages of young adults consume sweetened beverages, which can result in excess calories [30]. Finally, there are high rates of alcohol consumption in this age group [11], which can result in excess calories consumed both from the calories contained in alcohol, and from increased disinhibition and eating that occurs after consuming alcohol. Examples of other issues covered include social eating, peer pressure/influences, and effective time management.

### Treatment Components Unique to SBT

#### Weighing prescription

Participants in the SBT group were instructed not to weigh themselves at home during the active treatment phase of the program. They were weighed by the intervention staff in private just before each of the weekly group meetings. After treatment ended, they were encouraged to weigh themselves weekly at home to continue to monitor their progress, but were reminded to place primary emphasis on their behaviors, not on their weight.

#### Maintenance plan

During the last group meeting, interventionists worked with participants to help them formulate goals and an action plan for the next month. Within the context of a relapse prevention lesson, they were encouraged to set specific goals, develop self- reinforcement strategies, and formulate an action plan to deal with any slips they might experience. Participants were offered an optional booster session at 14 weeks to check in and reassess their goals.

### Treatment Components Unique to BSR

#### Weighing Prescription

Participants in the BSR group were given digital memory scales that stored up to 31 days of weight information and instructed to weigh themselves at home each day using this scale. They were told to weigh at the same time each day – ideally, first thing in the morning, just after waking and without clothes. Within a self-regulation framework, participants were taught to use the scale much like one would use a blood glucose monitor. That is, they learned to look for patterns and fluctuations in their weight and used the information to determine whether adjustments in energy-balance behaviors were necessary.

#### Education in self-regulation and color zone system

Participants in the BSR group received education on the principles of self-regulation [31], and its previous applications to diabetes [10] and weight management [9]. Within this framework, they were taught to use the scale to self-regulate their eating and activity behaviors. Based on their weekly weight, they compared their progress to their goal and used a color zone system to determine the appropriate course of action.

The color zone system was based on the successful maintenance model of STOP Regain [9], but modified for use in a weight loss program. Specifically, participants were considered to be in the green zone if their weight loss for the week was ≥ 1 kg or their weight loss overall averaged 1 kg per week. Each week they were in the green zone they received a small green gift (e.g., green gum, green tea) to provide positive reinforcement and to model the reinforcement skills taught in class. Participants were in the yellow zone if their weight loss was less than 1 kg from the week before (unless their overall weight loss averaged over 1 kg/week), and were instructed to use problem-solving skills to return to the green zone. Participants who gained weight from the previous week or who remained at the same weight for two weeks in a row (unless they already reached their goal weight) were in the red zone and were encouraged to make additional changes to affect energy balance (e.g., increasing intensity of physical activity, using a meal replacement for one meal).

#### Maintenance plan

During the last group meeting, interventionists worked with participants to help them formulate goals and an action plan for the next month. Within the context of a relapse prevention lesson, they were encouraged to formulate an action plan to deal with any slips they might experience. Participants also were introduced to the concept of *maintenance *color zones, and encouraged to use these new color zones to evaluate their weekly progress once they had met their weight loss goal. For maintenance, the green zone was defined as being within 1 kg of their weight at the end of the program; yellow zone was defined as 1.4 kg – 1.8 kg above goal/ending weight; and red zone was defined as 2.3 kg or more above their goal/ending weight. They were given instructions regarding appropriate actions to take when in each of the color zones, and were encouraged to practice self-reinforcement when in the green zone. Participants were offered an optional booster session at 14 weeks to check in and reassess their goals.

### Measures

#### Weight

Height and weight were measured at baseline, and BMI was calculated to determine eligibility for the current study. Weight was measured objectively at baseline, post-treatment and follow-up visits, as well as before each weekly group meeting.

#### Frequency of Weighing

Participants in both groups were asked to self-report frequency of self-weighing at each time point by responding to the following question: *"During the past month, how often did you weigh yourself?" *They were given the following response options: 1) several times a day, 2) one time each day, 3) several times a week, 4) one time a week, 5) less than once a week, 6) less than once a month, 7) never weighed myself. As an objective measure of frequency of self-weighing, participants in the BSR group were required to bring the digital memory scale provided to them to clinic after 1 month in the program and at post-treatment and follow-up.

#### Disordered Eating

Participants in both groups were asked to complete the Eating Disorder Examination-Self-Report Questionnaire (EDE-Q) at all time points to assess disordered eating behavior. The EDE-Q is a 38-item measure adapted from the Eating Disorder Examination (EDE), which is a structured clinical interview assessing the key behavioral features and associated psychopathology of eating disorders. The EDE-Q has excellent internal consistency and 2-week test-retest reliability for the four subscales of the EDE-Q: Restraint, Weight Concern, Shape Concern, and Eating Concern [32,33].

#### Body Image

Body image was assessed at all time points using the Body Shape Questionnaire, a 34-item scale that measures concerns about body shape and feelings of fatness. The Body Shape Questionnaire has been demonstrated to have adequate discriminant validity and a 3-week test-retest reliability of .88 [34,35].

#### Depression

Depression was assessed at all time points using the Beck Depression Inventory (BDI-II), a questionnaire designed to assess the cognitive, behavioral, affective, and somatic symptoms associated with depression [36]. Each of the 21 items asks participants to respond on a 4-point scale from 0 to 3, with total scores ranging from 0 (minimal) to 63 (severe). Previous studies have reported internal consistency coefficients ranging from .92 to .93 and a test-retest reliability of .93; it has been validated as a measure of depression [37], and is commonly used for clinical and research purposes.

### Statistical Analyses

The primary outcome of interest was weight change at follow-up. An a priori power analysis indicated that with a conservative estimate of correlation among measures (i.e., 0.5) and a significance level of 0.05, a sample size of 36 participants would provide 90% power to detect a medium within group effect in weight change over time. Primary analyses were conducted using general linear modeling in SPSS, Version 14. Initial chi-square analyses found no significant between-group differences on demographic variables of interest (i.e., sex, race, ethnicity); and an initial one-way ANOVA on baseline weight was also non-significant. To assess the effects of treatment on weight loss and psychological variables, repeated measures ANOVAS were conducted with weight evaluated at three time points (i.e., baseline, post-treatment and follow-up) and the between-subjects factor as treatment condition (i.e., behavioral self-regulation or standard behavioral). The effects of interest were the main effect of time and the interaction of time × group. Main outcome analyses were conducted using the intent to treat principle and missing weight data was imputed using the baseline carried forward method.

## Results

### Attendance and Retention

Attendance was excellent throughout the intervention, with no significant differences between groups. The mean number of sessions attended was 8.7 out of 10 (range 3–10) in the BSR group, and 8.6 out of 10 (range 1–10) in the SBT group. Retention rates were very strong across groups with a total of 93% of participants completing the post-treatment assessments, and 88% completing the follow-up assessment visit at 20 weeks, with no differences between groups.

### Adherence to Self-Weighing Prescription

At baseline, 5% of participants reported weighing more than once per day; 10% reported weighing daily, 25% reported weighing at least weekly, 20% reported weighing less than once a week, 20% reported weighing less than once a month, the remaining 20% reported never weighing themselves. The frequency with which participants weighed themselves was successfully manipulated by the intervention, as evidenced by no baseline differences between groups (p = .69) and significant differences at post-treatment and follow-up (*p *< .001).

At post-treatment 95% of participants in the BSR condition reported weighing daily. Two scheduled scale checks during the treatment phase confirmed these self-report data for the BSR group; 100% of participants at one-month and 95% of participants at post-treatment had the most recent 31 measurements stored in the digital memory scales. Further, at follow-up, 70.6% of participants in the BSR group reported continuing to weigh themselves daily.

In contrast, at post-treatment 94% of participants in the SBT condition reported adhering to the prescription they received to weigh only weekly at group meetings. At follow-up however, only 17.6% of participants in the SBT group reported continuing to weigh themselves weekly. Of note, at follow-up no SBT participants reported weighing themselves daily, 11.8% of participants reported weighing several times a week, and the remaining 70.6% of SBT participants reported weighing less than once a week.

### Weight Loss

**Figure 2:**
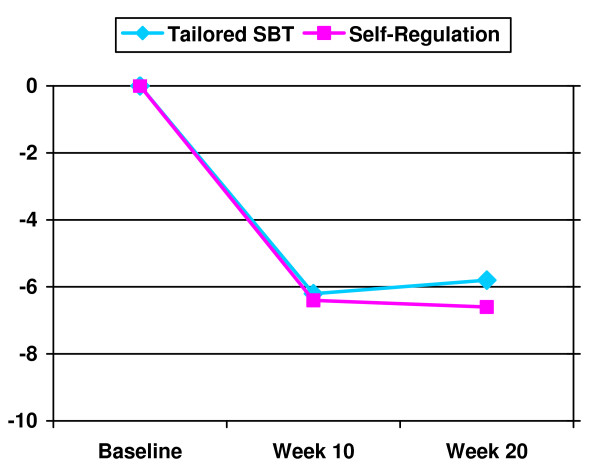
**Weight Change in Kilograms over Time**.

**Figure 3:**
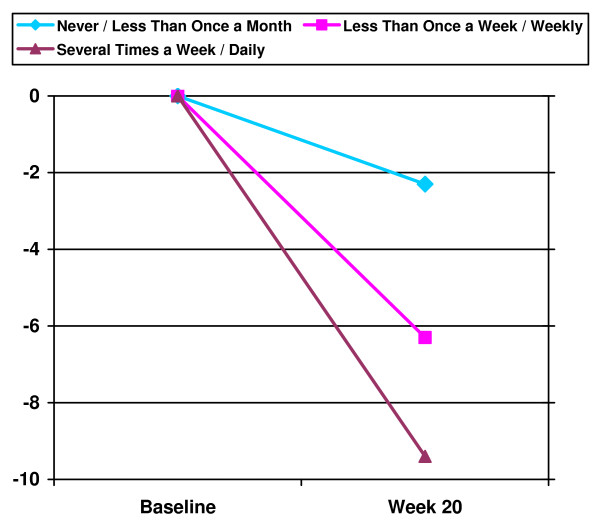
**Relationship between Frequency of Weighing at Follow-Up and Overall Weight Change across Treatment Groups**.

Both groups achieved significant weight losses at post-treatment [BSR = -6.4 kg (4.0); SBT = -6.2 kg (4.5)] and follow-up [BSR = -6.6 kg (5.5); SBT = -5.8 kg (5.2)], *F *(2, 76) = 57.010, *p *< .001; but the interaction of time × group was not significant, *F *(2, 76) = .170, *p *= .84, indicating no statistically significant effect of treatment group at post-treatment or follow-up. Of note, the BSR group not only maintained weight losses at follow-up, but mean weight losses improved in this group by -0.18 kg, compared to a mean regain in the SBT group of +0.37 kg, but this difference was not significant with the small sample size in this study (*p *= .52; see Figure 2). Further, although also non-significant with the current sample size, among those participants who completed the follow-up visit, 72% of participants in the BSR group maintained ≥99% of their initial weight loss at follow-up, whereas only 47% of participants in the SBT group maintained at this rate (*p *= .22). Across groups there was a positive association between frequency of weighing at follow-up and greater overall weight change from baseline to follow-up [r = .436, *p *= .01 (see Figure 3)].

### Impact on Psychological Variables

Overall, there was no evidence of negative psychological effects of daily weighing (see Table 1). In fact, participants in both conditions experienced significant positive changes on all parameters, including body image dissatisfaction, binge eating behavior, depressive symptoms, and all four subscales of the EDE-Q. As predicted, the interaction of time × group was not significant for any of these variables, indicating no effect of treatment condition.

**Table 1:** Means and standard deviations on psychological variables of interest

	Baseline		Post-Treatment (10 weeks)		Follow Up (20 weeks)		Time Effect		Interaction	
	BSR	SBT	BSR	SBT	BSR	SBT	*F*	*p*	*F*	*p*

BDI-II	9.8(7.9)	8.9(7.6)	3.3 (5.9)	4.7(3.7)	4.7(6.7)	5.3(4.6)	11.71	< .001	0.47	.63
BSQ	101.1(37)	104.8(26.8)	80.1(33.8)	76.5(21.1)	74.7(33.8)	78.6(31.0)	21.02	< .001	0.41	.65
Number of binge episodes	0.4(0.5)	0.7(0.5)	0.1(0.3)	0.3(0.5)	0.4(0.5)	0.3(0.5)	8.93	< .001	1.59	.21
EDE-Q Restraint	1.5(1.2)	1.7(0.9)	3.0(1.0)	3.3(1.0)	2.3(1.2)	2.4(0.6)	34.80	< .001	0.08	.92
EDE-Q Eating Concerns	1.4(1.2)	1.3(0.9)	0.7(0.6)	0.9(1.1)	1.0(1.0)	0.9(0.8)	7.13	< .001	0.73	.49
EDE-Q Weight Concerns	3.1(1.1)	3.3(0.8)	2.7(0.9)	2.6(0.7)	2.2(1.2)	2.2(0.6)	26.05	< .001	1.26	.29
EDE-Q Shape Concerns	3.6(1.4)	3.7(1.1)	2.5(1.2)	2.3(1.1)	2.4(1.6)	2.2(1.0)	28.41	< .001	0.41	.67

*Note: Statistics displayed are based on repeated measures analyses. Time effects also were significant from baseline to both post-treatment and follow-up (statistics not shown).

### Participant Feedback Data

After finishing the program, participants completed feedback forms about their experience in the Live Well program, as well as their preferences for format and duration of a weight loss program. Generally speaking, participants felt positively about the program, with 97% of respondents indicating that they would refer a friend to Live Well. Eighty-four percent of participants indicated that the 10-week program was sufficient in length for a weight loss trial targeting young adults. And while 86% percent of participants reported that they would be interested in a 12-month trial, all but one of those participants indicated that they would not have a desire to meet weekly beyond 10–12 weeks. Finally, when asked about the "ideal" format for an intervention, nearly half of respondents indicated they would prefer a program that used both face-to-face contact as well as the internet, and all but two of the remaining participants indicated they would prefer entirely face-to-face intervention delivery. Two participants indicated a preference for a combination of face-to-face and conference calls; no participants indicated that their preference would be an entirely internet-based program. There were no notable differences in feedback between treatment groups.

## Discussion

To our knowledge, this is the first study to recruit young adults for a weight loss program specifically tailored to this age group. Given the physical and public health consequences associated with weight gain and obesity during these years, effective intervention with young adults is imperative. Yet, very few young adults typically present for standard programs [5] and to date, no interventions have been designed to meet the unique needs of this population. Thus, the current findings offer important and novel information about treatment development for this age group. First, these data indicate that young adults can be recruited and retained in weight loss treatment, and that significant weight losses can be achieved through a brief intervention. Indeed, the weight losses achieved at post-treatment [BSR = -6.4 kg (4.0); SBT = -6.2 kg (4.5)] are far greater than the weight losses typically achieved by young adults after 6 months in standard programs [5], and participants in both groups maintained weight losses of greater than 6% of their initial body weight at follow-up (> 6.5% in SBT and > 7% in BSR). Moreover, these weight losses were maintained after a follow-up period that was as long as the intervention itself. The present findings suggest that relatively simple modifications of standard care may improve treatment with this age group relative to existing programs. Specifically, the homogeneity of groups may be an important factor. That is, it may be important both for recruitment and treatment success that group members are closer in age and that lessons reflect issues and problematic behaviors commonly experienced during these years.

An important aim of the current pilot study was to test the preliminary efficacy of a self-regulation program using daily self-weighing with this age group. We found that participants were willing to follow the self-weighing prescriptions in both conditions; and although there were no differences between groups for mean weight loss at post-treatment or follow-up, 72% of participants in the BSR group maintained ≥ 99% of their initial weight loss compared to only 47% of those in the SBT condition. Although not statistically significant with the small sample size in the current study, this does suggest some potential benefit of the self-regulation intervention over the long-term. Further, across treatment groups it appears that frequency of weighing after treatment ended was positively related to overall weight losses. Moreover, we found no evidence that daily self-weighing is associated with adverse psychological outcomes in this age group. In fact, to the contrary, participants in both treatment arms experienced positive changes on all parameters measured. Thus, consistent with previous data [6-9], these findings support the importance of frequent weighing for effective long-term weight control. Given the importance of self-monitoring for successful weight loss and maintenance, and the problems with maintaining self-monitoring of dietary intake and exercise in the long-term, future work should seek to determine whether daily self-weighing may be more successfully maintained than traditional forms of self-monitoring, and whether this is associated with improved weight loss maintenance in this population over longer-term follow-up. This may be of particular relevance for young adults striving to maintain weight losses achieved through a brief intervention similar to the one used in the present study.

The current study also provides novel data regarding format and duration preferences of this age group. Participant feedback data suggest that utilizing an alternative format for longer-term contact may be warranted. Despite the wealth of data to indicate that extending face-to-face contact improves long-term outcomes with adults, it is imperative that we think outside the box and create a format that meets the needs of this high-risk population that traditionally does not present for treatment. Given the significant time demands and transitions associated with this age group, it is unrealistic to expect that standard weight loss trials, consisting of 18-months of face-to-face contact will successfully reach this group. Indeed, available evidence suggests that such programs are neither appealing (e.g., problems with recruitment) nor effective (e.g., problems with retention and less impressive weight losses) in reaching young adults. Given the promising weight losses achieved through a brief face-to-face intervention in this sample, coupled with the feedback data indicating a lack of desire to meet weekly 10–12 weeks, exploring alternatives for extended contact (e.g., internet, conference calls) in future studies appears warranted.

While the current findings provide valuable new information, there are limitations that should be taken into account. First, this is an initial pilot study with a small sample and only 20-week follow-up. Thus, despite clear promise, the longer-term impact of the intervention remains unknown. Given the significant weight losses achieved, it appears that a fully powered trial comparing these interventions over a longer time period is warranted. Second, the current sample is predominantly female (85%), and as such, the application of these findings to males remains tentative. However, this is a consistent problem in weight control studies, and baseline analyses yielded no significant differences between groups on demographic variables, including gender. Third, the present study included individuals between 21 and 35 years of age, which represents a rather broad developmental period and also likely excludes many college students. Recently researchers have begun to argue that the late teens and early twenties should be considered a distinct period of the life course, often referred to as "emerging adulthood" [38]. It is possible that the weight control needs of this age group are distinct from those young adults in their late twenties and early thirties; as such, it may be beneficial to study these groups separately in future work. Finally, participants were young adults who responded to our ads and were willing to participate in an entirely face-to-face program for 10 weeks, and therefore may represent a unique subset of this age group. Nonetheless, a substantial strength of the current study is that it is the first to attempt to recruit and treat young adults in a weight loss program specifically tailored to the needs of this age group. This is a particularly high-risk population, and the retention rates and weight losses achieved are quite promising, particularly given the length of the follow-up period was identical to the intervention itself.

## Conclusion

In sum, the current pilot study suggests that targeting young adults specifically and tailoring program content and format to meet the unique needs of this age group may be an effective method of recruiting and retaining this population in weight control studies. Further, data support that frequent self-weighing may be an important, simple technique that can be used to improve long-term weight losses. Future studies should seek to replicate these findings with larger samples and longer-term follow-up. Additional research is also needed to determine the barriers to treatment, the most effective recruitment strategies, and the preferred duration and format for programs within diverse young adult populations.

## Competing interests

The authors declare that they have no competing interests.

## Authors' contributions

JGL participated in the design and coordination, carried out the intervention, performed the statistical analyses, and drafted the manuscript. AAG participated in the design and coordination and provided feedback on the manuscript. RRW conceived the study, participated in the design and provided feedback on the manuscript. All authors read and approved the final manuscript.
